# Application of generative adversarial networks (GAN) for ophthalmology image domains: a survey

**DOI:** 10.1186/s40662-022-00277-3

**Published:** 2022-02-02

**Authors:** Aram You, Jin Kuk Kim, Ik Hee Ryu, Tae Keun Yoo

**Affiliations:** 1 School of Architecture, Kumoh National Institute of Technology, Gumi, Gyeongbuk South Korea; 2 B&VIIT Eye Center, Seoul, South Korea; 3 VISUWORKS, Seoul, South Korea; 4 Department of Ophthalmology, Aerospace Medical Center, Republic of Korea Air Force, 635 Danjae-ro, Namil-myeon, Cheongwon-gun, Cheongju, Chungcheongbuk-do 363-849 South Korea

**Keywords:** Generative adversarial network, Ophthalmology image, Deep learning, Data augmentation, Domain transfer

## Abstract

**Background:**

Recent advances in deep learning techniques have led to improved diagnostic abilities in ophthalmology. A generative adversarial network (GAN), which consists of two competing types of deep neural networks, including a generator and a discriminator, has demonstrated remarkable performance in image synthesis and image-to-image translation. The adoption of GAN for medical imaging is increasing for image generation and translation, but it is not familiar to researchers in the field of ophthalmology. In this work, we present a literature review on the application of GAN in ophthalmology image domains to discuss important contributions and to identify potential future research directions.

**Methods:**

We performed a survey on studies using GAN published before June 2021 only, and we introduced various applications of GAN in ophthalmology image domains. The search identified 48 peer-reviewed papers in the final review. The type of GAN used in the analysis, task, imaging domain, and the outcome were collected to verify the usefulness of the GAN.

**Results:**

In ophthalmology image domains, GAN can perform segmentation, data augmentation, denoising, domain transfer, super-resolution, post-intervention prediction, and feature extraction. GAN techniques have established an extension of datasets and modalities in ophthalmology. GAN has several limitations, such as mode collapse, spatial deformities, unintended changes, and the generation of high-frequency noises and artifacts of checkerboard patterns.

**Conclusions:**

The use of GAN has benefited the various tasks in ophthalmology image domains. Based on our observations, the adoption of GAN in ophthalmology is still in a very early stage of clinical validation compared with deep learning classification techniques because several problems need to be overcome for practical use. However, the proper selection of the GAN technique and statistical modeling of ocular imaging will greatly improve the performance of each image analysis. Finally, this survey would enable researchers to access the appropriate GAN technique to maximize the potential of ophthalmology datasets for deep learning research.

## Background

Over the past decades, ophthalmologic images have gained a huge interest because of their importance in healthcare to prevent blindness due to ocular diseases and to decrease their socioeconomic burden worldwide [1]. In particular, the widespread availability of fundus photography and optical coherence tomography (OCT) provides an opportunity for early detection of diabetic retinopathy, age-related macular degeneration, and glaucoma [2]. However, there are still many constraints on the use of data-driven artificial intelligence (AI) models in ophthalmology. Ocular imaging data have been expanding globally [3], but there is a shortage of high-quality images and pathological data from patients to train AI models [4]. In addition, there are many diagnostic ocular imaging modalities such as color fundus photography, retinal OCT, ultra-widefield fundus photography, retinal angiography, ultrasonography, corneal tomography, and anterior segment OCT. Because imaging techniques differ from each other in terms of structural complexity and image dimensionality, the need for cross-modality image processing methods has increased to improve the disease prediction models.

In recent years, generative adversarial network (GAN) has become the technique of choice for image generation and translation in the field of medical imaging [5]. GAN, which is a new type of deep learning developed by Ian Goodfellow [6], can automatically synthesize medical images by learning the mapping function from an arbitrary distribution to the observed data distribution, which is the process of extracting mathematical relationships from data distributions for matching input to output data. As deep learning requires more data to build better accurate models, the medical research community requires more databases from various imaging modalities such as computed tomography (CT) and magnetic resonance imaging (MRI) [7]. Accordingly, many experiments have been performed to demonstrate the benefit of using GAN, which can generate realistic synthetic images. Previous publications in radiology have shown that the application of GAN includes data augmentation, denoising, super-resolution, domain transfer between modalities, and segmentation [8]. They demonstrated that the potential gains from GAN can improve deep learning models for pathological conditions and can support clinicians for diagnosis using complex medical images. While medical image processing using GAN has been actively studied in radiology [9], there have been relatively few studies using GAN in the field of ophthalmology image domains.

A previous literature review showed that the adoption of GAN for medical imaging is increasing rapidly [10], but it is not familiar to researchers in the field of ophthalmology. Since ophthalmology imaging techniques are becoming more important for diagnosing ocular diseases, the use of GAN will gradually increase to achieve a more accurate diagnosis. In this work, we present a literature review on the application of GAN in ophthalmology image domains to discuss important contributions and identify potential future research directions. This paper attempts to guide future research on image processing in the ophthalmic domain through the proper application of GAN techniques.

## Review

### Overview

We detail the studies using GAN for ophthalmology image domains from available literature. We summarized how GAN was utilized in the field of ophthalmology imaging. The type of GAN used in the analysis, task, imaging domain, and outcome of the application of GAN were collected to verify its usefulness. We searched for potentially relevant literature in PubMed, Embase, and Google Scholar databases using the following search strategy: ((generative adversarial network) OR (GAN) OR (deep generative model)) AND ((ophthalmology) OR (diabetic retinopathy) OR (age-related macular degeneration) OR (fundus photography) OR (optical coherence tomography)). The initial selection of studies was performed based on texts with titles and abstracts. We included only peer-reviewed articles published before June 2021. Articles that did not contain original research using GAN were excluded. Only articles written in English were included in the study, and studies without a specific description of the GAN model were excluded. In the case of multiple publications of the same research, we regarded them as one study. The initial search yielded 855 articles. Of these, 806 were removed because their studies or manuscripts were not related to either GAN or ophthalmology images. A further three were excluded because they were duplicates of other studies in the list or were not research articles. Finally, 48 articles were included in the final literature review. To limit bias in the search, additional searches were performed using other common ocular diseases (such as dry eye, conjunctivitis, cataract, glaucoma, retinal vein occlusion, central serous chorioretinopathy, and strabismus) and other imaging modalities found in the previous search. However, no additional research articles were obtained.

Since a comprehensive list of ophthalmology image datasets has been provided in previous studies [3, 11], we found no reason to discuss ocular image datasets in this work. Traditional data augmentation refers to an increase in the number of training examples through the rotation, flipping, cropping, translation, and scaling of existing images to improve the performance of deep learning models, and can also be used to train GAN models. Generally, most GAN techniques rely on large amounts of annotated data, although several studies have targeted data augmentation in a small amount of pathological data.

### GAN techniques

**Fig. 1:**
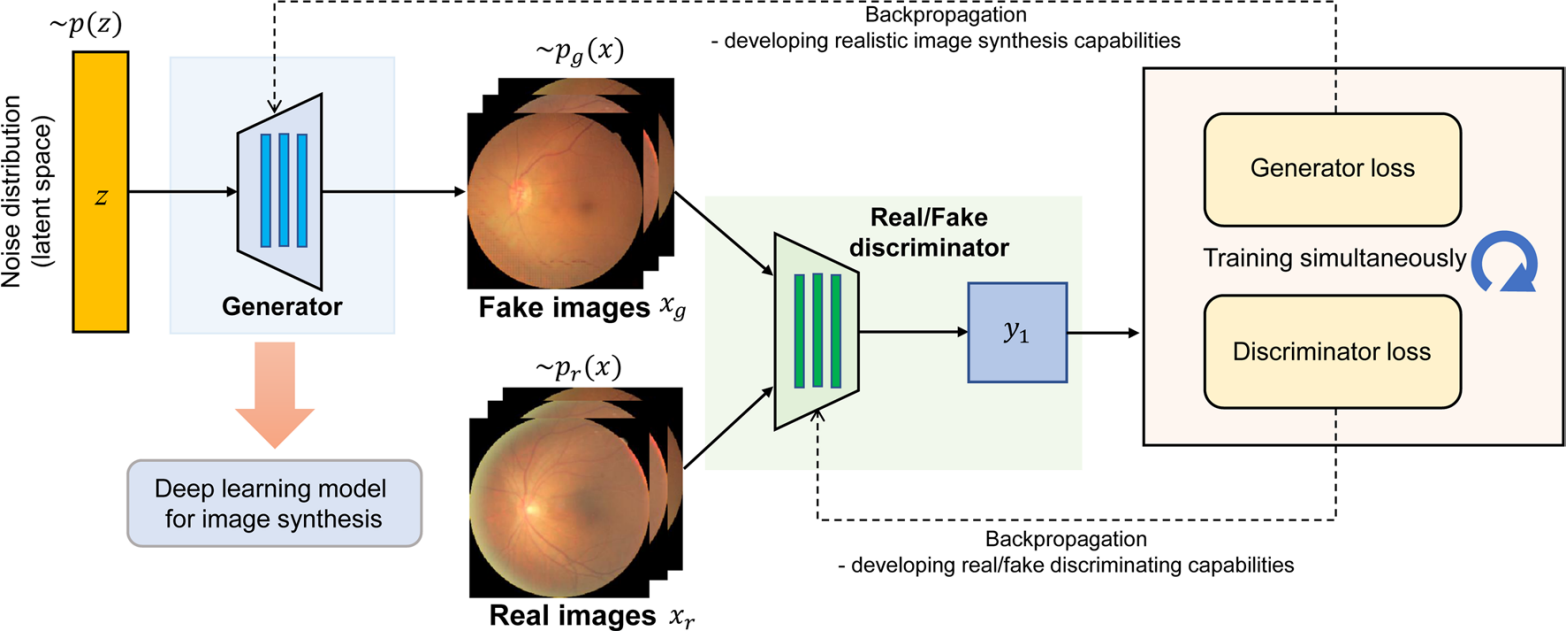
An illustration of a basic architecture of GAN (vanilla GAN) for retinal image synthesis. The generator transforms a noise vector $z$ from the distribution $p(z)$ into a synthesized retinal image ${x}_{g}$. The discriminator distinguishes the synthetic and real retinal images based on the distributions of ${x}_{g}$ and ${x}_{r}$, respectively. The generated image samples form a distribution ${p}_{g}(x)$, which is desired to be an approximation of ${p}_{r}(x)$ from real image sample, after successful training

This section provides the general concepts of GAN for analyzing ophthalmology images, especially the architectures most frequently encountered in the literature reviewed. The basic structure of a GAN is called a vanilla GAN [6]. The architecture of the vanilla GAN consists of two separate deep learning models, including the generator, which synthesizes candidate samples based on the data distribution of the original dataset, and a discriminator, which tries to distinguish the synthesized candidate samples from the real samples from the original dataset. These two modules are trained simultaneously because the gradient information is back-propagated to the generator to increase realistic image synthesis capabilities and to the discriminator to increase real/fake discriminating capabilities. After vanilla GAN was introduced, GAN was highlighted because of its ability to generate realistic synthetic images based on the original dataset. Figure 1 illustrates the structure of the vanilla GAN and retinal images generated. In the example of retinal image synthesis, vectors of late space are randomly selected initially; however, after training, they obtain an appropriate functional relationship with the generated image. Randomly generated images and original real retinal images are classified by the discriminator and this result is back-propagated and reflected in the training of both the generator and the discriminator. Finally, the desired outcome after training the GAN is that the pixel distributions from the generated retinal images should approximate the distribution of real original retinal images. According to the original paper describing GAN, the generator is like a team that produces counterfeit money, which wants to use counterfeit money without it being detected, and the discriminator is like the police detecting counterfeit money. The competition between the two teams results in better counterfeiting [6]. After image generation using GAN was popularized, novel GAN techniques were constantly developed in the machine learning community. In this review, we found that most studies on ophthalmology images have used progressively growing GAN (PGGAN), conditional GAN, Pix2pix, and cycle-consistent GAN (CycleGAN).

Currently, vanilla GAN is not widely used because of its low-quality outputs and instability during training. However, it has been the basis of recent GAN variant techniques (Table 1). A deep convolutional GAN (DCGAN) is based on the vanilla GAN by replacing the building block with fully convolutional layers [10]. Wasserstein GAN is an improved version of the vanilla GAN that uses a metric of the distance between two probability distributions (Wasserstein distance) as a loss function [12]. PGGAN is an extension of the vanilla GAN with a progressively growing generator and discriminator to generate realistic high-resolution images. The main concept of PGGAN is to build generators and discriminators, starting from a low-resolution to a high-resolution network. The newly added layers model fine-grained details as the training progresses. As the images are generated from a random noise vector, the original PGGAN cannot generate new instances with objects in the desired condition. Additionally, StyleGAN is a variant of PGGAN that adds the style transfer function in a conditional setting to the architecture of PGGAN [13]. Style and feature changes in synthetic images can be performed using an additional mapping network for the latent space in the generator of StyleGAN.

**Table 1:** The characteristics of typical GAN variant techniques and examples of general tasks in general medicine and ophthalmology fields

GAN Techniques	Dataset	Characteristics	Task examples in general medicine	Task examples in ophthalmology
Deep convolutional GAN (DCGAN)	Images from one domain	Improved image quality using deep convolutional layers	Augmentation of CT images [88]	Fundus photographs synthesis [39]
Wasserstein GAN (WGAN)	Images from one domain	Using Wasserstein distance as a loss function	Augmentation of CT images [89] Removing artifacts in CT [90]	Anomaly detection [25] OCT segmentation [91]
Progressively growing GAN (PGGAN)	Images from one domain (generally high resolution)	High resolution & realistic image generation	X-ray image synthesis [92] Data augmentation for cytological images [93]	Data augmentation for fundus photography, retinal OCT, and ocular images [40, 46, 82] Super-resolution of fundus photographs [55]
StyleGAN	Images of one domain or multiple domains (unpaired images)	Disentanglement of representations (mapping features to low dimensions)	Augmentation of CT and MRI images in specific conditions [13, 94] Skin image synthesis [95]	None
Conditional GAN (vector input models)	Images annotated by conditional variables	Image synthesis conditioned to specific variables	Super-resolution guided by a conditional variable [96]	Data augmentation for retinal OCT [44] Post-intervention (orbital decompression) prediction [15]
Conditional GAN (Pix2pix and other image input models)	Paired images of two domains or classes. (Training samples should be aligned)	Supervised learning for image-to-image translation	Super-resolution for fluorescence microscopy images [97] Domain transfer (CT → PET) [98] Segmentation of lungs from chest X-ray [99] CT image synthesis [100]	Domain transfer (fundus photography → angiography) [62] Retinal OCT segmentation [36] Retinal vessel segmentation [28] Data augmentation for fundus photography and corneal topography [17, 47]
Super-resolution GAN (SRGAN)	Low- and high-resolution image pairs	Adopting perceptual loss to generate super-resolved realistic images	Super-resolution for dental X-ray [101]	Super-resolution for optic disc photography [56]
Cycle-consistent GAN (CycleGAN)	Unpaired images of two domains or classes	Adopting a cycle consistency for domain transfer without any paired dataset	Manipulating breast imaging [102] Data augmentation for CT and throat images [103, 104] Segmentation for cardiac ultrasound [105]	Denoising for fundus photography and OCT [22, 53] Domain transfer (Ultra-widefield retinal images → classic fundus photography) [63]
StarGAN	Unpaired images of multiple domains or classes	A single network to achieve translation of multiple domains	Domain transfer between MRI contrasts [24]	None

*CT* = computed tomography; *GAN* = generative adversarial network; *MRI* = magnetic resonance imaging; *OCT* = optical coherence tomography; *PET* = positron emission tomography

In many cases, synthetic images should be generated with the desired properties to adopt GAN for medical purposes. A conditional GAN is an extended architecture of vanilla GAN, where both the generator and discriminator are trained using not only the original dataset but also additional conditioning variables [14]. To achieve good image generation performance in multiple domains, researchers have modified the generators of conditional GAN in various deep learning architectures. Currently, conditional GAN includes many types of GAN models because the condition variable can be any variable including a single status variable [15], images of the same or different domains [16], masked images [17], and guided heatmap images [18]. If the conditional variable is set as an image, the training dataset should commonly contain aligned image pairs. The most widely used form of conditional GAN is Pix2pix, which contains an image-to-image translation framework [19]. Instead of using a conventional encoder-decoder as a generator, Pix2pix adopts a U-Net-like architecture with skip connections to generate synthetic images from the input images. In Pix2pix, the discriminator is used at the local image patch level to improve the performance. The GAN architecture for a super-resolution task (SRGAN) was developed by adopting a conditional GAN and perceptual loss [20]. However, conditional GAN models, including Pix2pix, have a critical disadvantage in that the shortage of paired datasets restricts their application to real problems.

Recently, CycleGAN was developed to generate images without matching paired images [21]. CycleGAN involves the simultaneous training of two generators and two discriminators. The CycleGAN adopts a cycle consistency, which is based on the idea that the output of the first generator can be used as input to the second generator, and the output of the second generator should be like the original image. This cycle consistency allows CycleGAN to learn the characteristics of the two image domains to transfer the domains without any paired dataset. The weights for the training parameters of CycleGAN modules can be tuned depending on the image domain or task. CycleGAN can perform denoising by mapping clean and noisy domains from unpaired training data [22]. Currently, variants of CycleGAN, such as StarGAN [23] and its variants [24], have been introduced to achieve high performance in a multiple domain transfer problem. The characteristics of typical GAN techniques and examples of general tasks in general medicine (especially radiology) and ophthalmology fields are summarized in Table 1. It should be noted that there are several cases where it is not classified as a specific type of GAN because the custom architectures of GAN were commonly designed for each imaging domain.

### Applications in ophthalmology

**Fig. 2:**
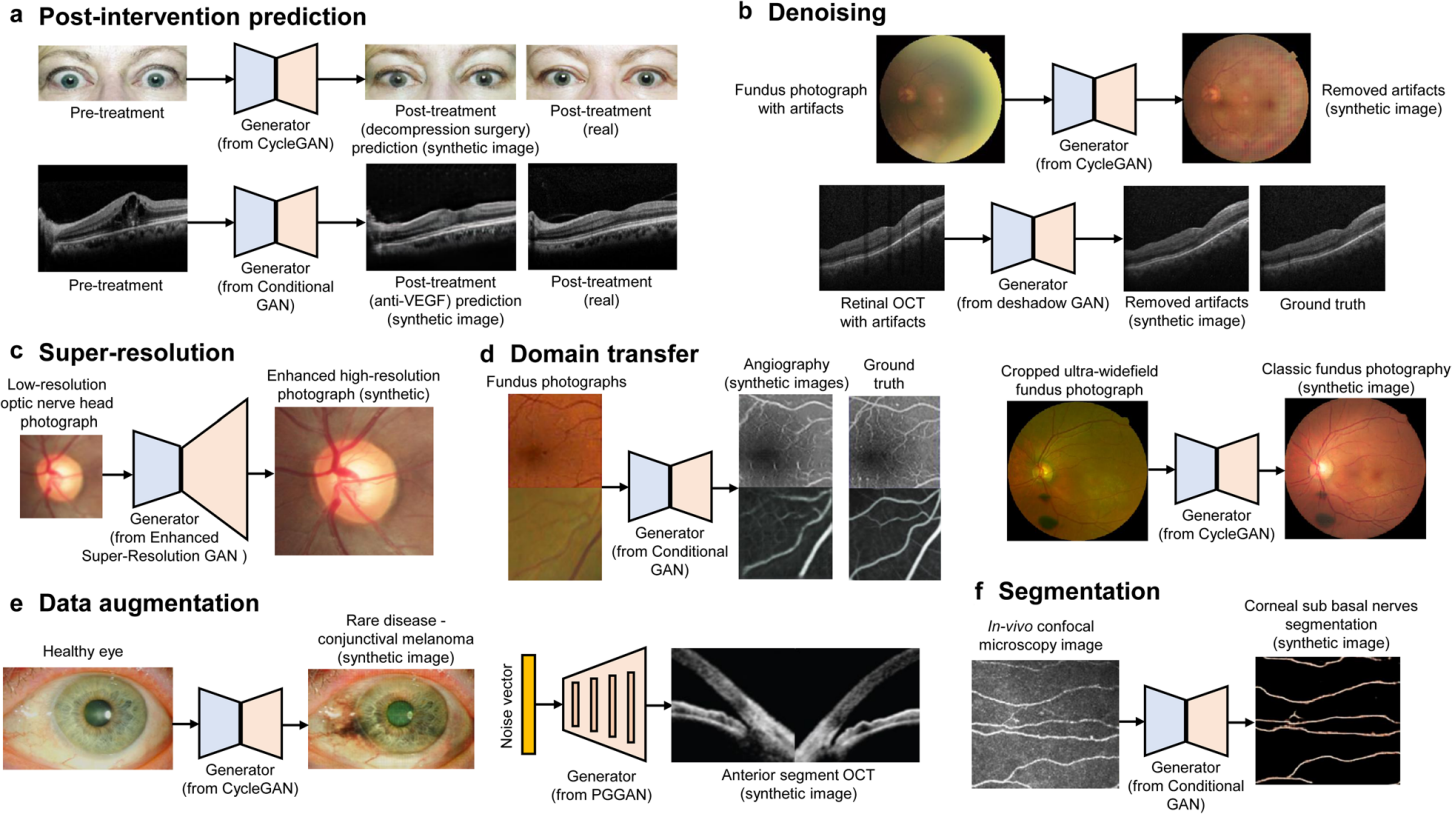
Examples of applications of GAN in ophthalmology image domains. **a** Post-intervention prediction for decompression surgery for thyroid ophthalmopathy [15] and anti-vascular endothelial growth factor (VEGF) therapy for neovascular age-related macular degeneration [66]. **b** Denoising in fundus photography [53] and peripapillary optical coherence tomography (OCT) [16]. **c** Super-resolution for optic nerve head photography [56]. **d** Domain transfer for fundus photography to angiography [62] and ultra-widefield to classic fundus photography (re-analysis in this work) [63]. **e** Data augmentation for ocular surface images [46] and anterior segment OCT [82]. **f** Segmentation for corneal sub basal nerves in in vivo confocal microscopy images [37]. Most images were generated according to publicly available datasets and the methods of each study (some cases are based on our own dataset)

**Fig. 3:**
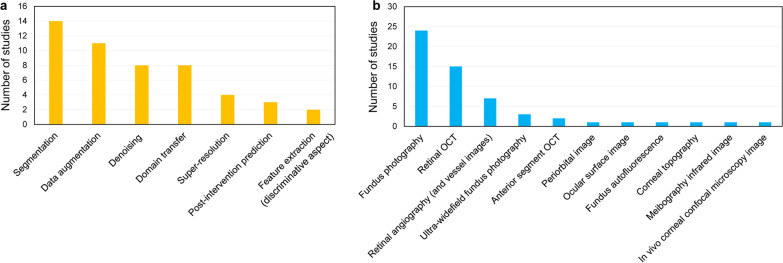
Number of studies that were reviewed in this work grouped according to tasks and image domains. **a** Study objectives in the application of GAN. **b** Ophthalmology image domains for the use of GAN. If one study deals with two issues, it was reviewed and double-counted appropriately

Here, we survey the literature on GAN for ophthalmology image domains. Applications are introduced according to the type of tasks of GAN models, including segmentation (15 studies), data augmentation (11 studies), denoising (8 studies), domain transfer (8 studies), super-resolution (4 studies, two studies overlap with denoising), post-intervention prediction (3 studies), and feature extraction (2 studies). Figure 2 shows examples of the applications of GAN. Figure 3 shows the number of studies based on the tasks of the GAN and image domains. Some studies that handled the two image domains were double-counted. Most studies have focused on the generative aspect of GAN, and only two studies, f-AnoGAN [25] and AMD-GAN [26] adopted the discriminative aspect with feature extraction. The survey showed that segmentation was the most studied task in GAN in ophthalmology. Among ophthalmology imaging domains, fundus photography (24 studies) has been most frequently analyzed using GAN in the literature. GAN has also been used in various imaging domains, including retinal OCT (15 studies), retinal angiography (7 studies), ultra-widefield fundus photography (scanning laser ophthalmoscopy, 3 studies), anterior segment OCT (two studies), periorbital facial image for orbital diseases (1 study), ocular surface image (1 study), corneal topography (1 study), meibography infrared imaging (1 study), and in vivo corneal confocal microscopy (1 study). If one study deals with two modalities, it was reviewed and double-counted if necessary. Although conditional GAN is most frequently mentioned in the survey, it is difficult to conclude that it has been most widely used because the conditional GAN models refer to a wide variety of deep learning structures for each study. A table detailing the literature is provided for each section of the application.

### Segmentation

Image segmentation is a task where pixels or areas in an image are assigned a category label. Segmentation is the most frequently studied (14 studies) focusing on the identification of structures such as retinal vessels, retinal layers, and optic nerve. Identifying pathological areas on the ocular images can help clinicians to diagnose more accurately, and thus segmentation is an important task for developing AI models for medicine. Table 2 shows a summary of the literature review for the segmentation task using GAN.

**Table 2:** Summary of literature review for image segmentation task using GAN in ophthalmology imaging domains

Publication	Basic technique	Domain	Target	Summary
Iqbal et al. [27]	Conditional GAN	Fundus photography	Retinal vessels	The framework achieved retinal vessels image segmentation from a small training set
Wang et al. [33]	Conditional GAN (using PatchGAN)	Fundus photography	Optic disc and optic cup	Unsupervised domain adaptation for joint optic disc and cup segmentation using a patch-based discriminator
Son et al. [28]	Conditional GAN (using U-Net as a generator)	Fundus photography	Retinal vessels	The GAN model segmented retinal vasculature and the optic disc using a dataset, which consisted of fundoscopic images and manual segmentation of the vessels
Rammy et al. [29]	Conditional GAN	Fundus photography	Retinal vessels	Patch-based GAN with additional loss function to learn thin and thick vessel segmented retinal vessels with the enhanced performance
Park et al. [30]	Conditional GAN	Fundus photography	Retinal vessels	The proposed GAN model with a multi-kernel pooling and false negative loss could segment retinal blood vessels more robustly
Heisler et al. [36]	Pix2Pix (conditional GAN)	Peripapillary retinal OCT	Nerve fiber layer, Bruch’s membrane, choroid-sclera boundary	The use of a generative adversarial network and unlabeled data can improve the performance of segmentation for the 3D morphometric analysis of glaucomatous optic nerve head volumes
Yang et al. [31]	Conditional GAN (topological structure-constrained)	Fundus photography	Retinal vessels	The topological structure-constrained proposed GAN model identified retinal arteries and veins via segmentation from the complex background of retinal images
Yang et al. [106]	Conditional GAN	Fundus photography	Retinal vessels	To separate blood vessels from fundus image, the model could detect more tiny vessels and locate the edge of blood vessels more accurately
Zhao et al. [32]	Conditional GAN (with a large receptive field)	Fundus photography	Retinal vessels	The proposed retinal vessel segmentation algorithm using GAN with a large receptive field could capture large-scale high-level semantic vessel features
Kadambi et al. [34]	Wasserstein GAN	Fundus photography	Optic disc and optic cup	WGAN-based domain adaptation showed a better performance than baseline models for the joint optic disc-and-cup segmentation in fundus images
Bian et al. [35]	Conditional GAN (using U-Net as a generator)	Fundus photography	Optic disc and optic cup	The proposed method successfully performed optic disc and cup segmentation. The cup-to-disc ratio was automatically calculated with a good performance
Khan et al. [38]	Conditional GAN	Meibography infrared images	Meibomian gland	The proposed GAN automatically identified the area of the meibomian glands and outperformed the state-of-art methods
Yildiz et al. [37]	Conditional GAN	In vivo corneal confocal microscopy images	Sub-basal nerves	Automatic segmentation of sub-basal nerves in in vivo confocal microscopy images was performed using the U-Net and GAN-based techniques as a diagnostic tool for corneal diseases
Zhou et al. [107]	Conditional GAN (using U-Net as a baseline architecture)	Fundus photography	Retinal vessels	The GAN model strengthened retinal vessel segmentation in the low-contrast background using a symmetric equilibrium GAN (U-Net-based), multi-scale features refine blocks and attention mechanism

*GAN* = generative adversarial network; *OCT* = optical coherence tomography; *WGAN* = Wasserstein GAN

GAN techniques are typically used to segment retinal vessels from fundus photographs. For decades, retinal vessel segmentation has been a challenging problem in the computer science community because vessels have various widths, colors, tortuosity, and branching. After conditional GAN was applied to this problem [27], many variants of the conditional GAN were proposed by modifying the architectures. In particular, Son et al. [28] improved the conditional GAN using a generator based on U-Net, similar to the Pix2pix architecture. To improve segmentation performance, some studies employed patch-based GAN [29], multi-kernel pooling layers [30], topological structure-constrained models [31], large receptive fields [32], and symmetric equilibrium generators with attention mechanisms [17]. Since most of these studies have been conducted using limited annotated vessel datasets without collaboration with ophthalmologists, validation with real patient data was not performed.

In addition, accurate cup-to-disc ratio calculation based on optic disc segmentation is an important problem for evaluating optic nerve damage and glaucoma. Damage to the optic nerve increases the cup-to-disc ratio, which is difficult to compare with the naked eye if the damage is small. In recent studies, GAN was useful for segmenting the optic disc and cup in fundus photographs using patch-based conditional GAN [33] and Wasserstein GAN [34]. The cup-to-disc ratio, a clinically used measurement to assess glaucoma progression, can be directly calculated from the optic cup and disc segmentation using conditional GAN [35]. This study showed comparable performance in assessing the cup-to-disc ratio for glaucoma screening.

Another application found in the literature is the segmentation of retinal layers in OCT images. Retinal layers consist of the vasculature, neurons, glia, and their connections, and each layer changes differently under pathological conditions. Pix2pix was successfully applied to segment the retinal nerve fiber layer, Bruch’s membrane, and choroid-sclera boundary in peripapillary retinal OCT images [36]. GAN was also applied to evaluate corneal pathological conditions using in vivo confocal microscopy images [37]. In this study, the segmentation of corneal sub basal nerves was achieved using a conditional GAN to detect corneal diseases. The meibomian gland can be evaluated by the GAN to segment the area of the meibomian glands in meibography infrared images [38]. In this study, the conditional GAN outperformed U-Net and masked regions with convolutional neural networks (mask R-CNN).

### Data augmentation

The development of a machine-learning model requires enough data. Imbalanced data is a barrier to the training model, and the lack of data often presents in many medical problems because of barriers to access and usability [3]. Traditional data augmentation is commonly unable to extrapolate the generated data, which leads to data bias and suboptimal performance of trained models. Many researchers have shown that data augmentation using GAN techniques can provide additional benefit over traditional methods [8]. Recently, GAN techniques have been widely used to synthesize realistic medical images for data augmentation. Here, 11 studies investigated data augmentation for ophthalmology imaging domains (see Table 3). Several studies using GAN have focused on fundus photography and retinal OCT image generation to augment training datasets for machine learning.

**Table 3:** Summary of literature review for data augmentation task using GAN in ophthalmology imaging domains

Publication	Basic technique	Domain	Summary
Diaz-Pinto et al. [39]	DCGAN	Peripapillary fundus photography (optic disc photo)	DCGAN was able to generate high-quality synthetic optic disc images
Burlina et al. [40]	PGGAN	Fundus photography	The GAN technique was used to synthesize high-resolution realistic fundus images serving as proxy data sets for use by retinal specialists and deep learning models
Zheng et al. [43]	PGGAN	Retinal OCT (spectral domain)	The image quality of real images *vs.* synthetic OCT images generated by GAN was similar; the synthetic OCT images were able to serve as augmentation of training datasets for deep learning models
Zhou et al. [17]	Conditional GAN	Fundus photography	To generate a large amount of balanced training data, the GAN model synthesized high-resolution diabetic retinopathy fundus images which can be manipulated with arbitrary grading and lesion information
Wang et al. [41]	Multi-channel GAN (modified vanilla GAN)	Fundus photography	The model generated a series of sub-fundus images corresponding to the scattering diabetic retinopathy features and made full use of both labeled and unlabeled data
He et al. [42]	Label smoothing GAN (modified vanilla GAN)	Retinal OCT	The GAN model generated the synthetic unlabeled images from limited OCT training samples, and the mixing of the synthetic images and real images can be used as training data to improve the classification performance
Yoo et al. [45]	CycleGAN	Retinal OCT	GAN generated OCT images of rare diseases from normal OCT images and increased the accuracy of diagnosing rare retinal diseases with few-shot classification
Kugelman et al. [44]	Conditional GAN	Retinal OCT (patch level)	GAN was feasible to generate patches that are visually indistinguishable from their real variants and improved the segmentation performance
Zheng et al. [82]	PGGAN	Anterior Segment OCT	The synthetic OCT images generated by GAN appeared to be of good quality, according to the glaucoma specialists, and the deep learning model for angle-closure detection was improved using both synthetic and real images
Yoo et al. [46]	CycleGAN, PGGAN	Ocular surface image	To improve the diagnostic accuracy, GAN was adopted to perform data augmentation of ocular surface images with conjunctival melanoma
Abdelmotaal et al. [47]	Pix2pix	Corneal topography (Scheimpflug images)	The synthesized images showed plausible subjectively- and objectively-assessed quality. Training deep learning with a combination of real and synthesized images showed better classification performance to detect keratoconus

*GAN* = generative adversarial network; *DCGAN* = deep convolutional GAN; *OCT* = optical coherence tomography; *PGGAN* = progressively growing GAN

In recent years, generating realistic fundus photographs has become a challenging issue. DCGAN was initially used to generate synthetic peripapillary fundus photographs [39]. The machine learning model based on DCGAN showed better diagnostic performance for glaucoma detection than conventional deep learning models. Burlina et al. evaluated the performance of a PGGAN to generate realistic retinal images [40]. In their study, two retinal specialists could not distinguish real images from synthetic images. Zhou et al. used a conditional GAN to generate high-resolution fundus photographs based on structural and lesion mask images [17]. The multi-channel generator technique, which trains multiple GAN models for each feature such as exudates, microaneurysms, and bleeding, was used to augment fundus photographs to overcome the imbalance of data to build a diabetic retinopathy detection model [41].

The generation of synthetic retinal OCT is another important task for data augmentation in the development of machine-learning models for automated OCT diagnosis. By integrating both normal and pathological data, GAN can generate synthetic OCT images with various pathological grades for data augmentation [42]. Zheng et al. showed that realistic retinal OCT images could be generated using PGGAN, which could improve the classification performance of deep learning models [43]. Data augmentation based on conditional GAN also improved the segmentation performance of retinal OCT images [44]. CycleGAN was applied to OCT data augmentation for rare retinal diseases in a few-shot learning system design [45]. GAN has been used for data augmentation of anterior OCT images for angle-closure glaucoma, ocular surface images for conjunctival disease [46] and corneal topography images for keratoconus detection [47].

### Denoising & super-resolution

Image enhancement tasks such as denoising and super-resolution are important because ophthalmology images generally suffer from limitations of the device, the skill of the examiner, variations of ocular anatomy, and transparency of the visual axis. Image quality may affect the diagnostic performance using ocular images although the device and software offer suppression of noise and artifacts. As each imaging domain has characteristic noise and artifacts, several research groups have tried to develop data-driven GAN models tailored to each domain. There are 10 studies investigating denoising or super-resolution for ophthalmology imaging domains (detailed in Table 4).

**Table 4:** Summary of literature review for image enhancement (denoising and super-resolution) tasks using GAN in ophthalmology imaging domains

Publication	Basic technique	Domain	Target	Summary
Halupka et al. [48]	Modified Wasserstein GAN + perceptual loss (conditional GAN)	Retinal OCT (spectral domain)	Removing speckle noise	The GAN was used to reduce speckle artifacts in retinal OCT images. The method improved the image quality metrics for OCT
Mahapatra et al. [55]	PGGAN with a conditional design	Fundus photography	Super-resolution	Image super-resolution using multi-stage PGGAN outperforms competing methods and baseline GANs. The super-resolved images can be used for landmark and pathology detection
Huang et al. [49]	Conditional GAN	Retinal OCT	Super-resolution and removing noise	The GAN model effectively suppressed speckle noise and super-resolved OCT images at different scales
Ouyang et al. [51]	Conditional GAN	Anterior Segment OCT	Removing speckle noise	The model removed undesired specular artifacts and speckle-noise patterns to improve the visualization of corneal and limbal OCT images
Yoo et al. [53]	CycleGAN	Fundus photography	Removing artifacts and noise	The GAN model removed the artifacts automatically in a fundus photograph without matching paired images
Cheong et al. [16]	DeshadowGAN (modified conditional GAN with perceptual loss)	Peripapillary retinal OCT (spectral domain)	Removing vessel shadow artifacts	The GAN model using manually masked artifact images and perceptual loss function removed blood vessel shadow artifacts from OCT images of the optic nerve head
Chen et al. [50]	Conditional GAN	Peripapillary retinal OCT (spectral domain)	Removing speckle noise	The GAN model was designed for speckle noise reduction in OCT images and preserved the textural details found in OCT
Das et al. [52]	CycleGAN	Retinal OCT	Super-resolution and removing noise	To achieve denoising and super-resolution, adversarial learning with cycle consistency was used without requiring aligned low–high resolution pairs
Ha et al. [56]	Enhanced super-resolution GAN (SRGAN)	Peripapillary fundus photography (optic disc photo)	Super-resolution	The GAN approach was capable of 4-times up-scaling and enhancement of anatomical details using contrast, color, and brightness improvement
Yuhao et al. [54]	CycleGAN	Fundus photography	Removing artifacts and noise	The developed model dehazed cataractous retinal images through unpaired clear retinal images and cataract images

*GAN* = generative adversarial network; *OCT* = optical coherence tomography; *PGGAN =* progressively growing GAN

To remove speckle noise in retinal OCT images, a conditional GAN model with Wasserstein distance and perceptual loss was proposed [48]. Huang et al. showed that both super-resolution and noise reduction can be performed simultaneously using a conditional GAN [49]. Cheong et al. built DeShadowGAN using manually masked artifact images and conditional GAN with perceptual loss and demonstrated the effectiveness of the model in removing shadow artifacts [16]. Similarly, conditional GAN has also been applied to remove speckle noise in peripapillary retinal OCT [50] and anterior segment OCT [51]. However, image denoising methods using conditional GAN can match low- and high-quality image pairs; however, these data are typically unavailable in the medical field. Therefore, Das et al. used CycleGAN for super-resolution and noise reduction in retinal OCT images to facilitate unpaired image datasets [52].

Fundus photography has several artifacts and noise, including overall haze, edge haze, arcs, and lashes. In a super-resolution problem, artificial manipulation to reduce image resolution is possible. Since it is difficult to collect paired clean and artifact fundus photographs, CycleGAN has been used to improve the image quality of fundus photography [53, 54]. These studies showed that CycleGAN can effectively reduce artifacts to provide clearer retinal images to clinicians. PGGAN was also shown with a conditional design that was applied to super-resolution fundus photography [55]. Ha et al. adopted that to generate high-resolution synthetic optic disc images with a 4-times up-scaling using SRGAN [56].

### Domain transfer

Most machine learning works have performed the development and validation of data from the same domain. To build a more generalized machine-learning model, data from different domains might be fused through domain transfer, which is the transfer between different imaging modalities. The domain transfer task of GAN is the cross-modality image synthesis process by which images are generated for one modality based on another. Cross-domain modality using the domain transfer technique has shown the possibility of obtaining additional clinical information without additional examinations [57]. Eight studies using GAN mainly focused on domain transfer for ophthalmology imaging domains (shown in Table 5). Notably, several studies that used image transfer generators were categorized as data augmentation tasks because they focused on image synthesis tasks. The concept of conditional GAN has been used in most studies because the generator must have an image input channel as a conditional variable for domain transfer. Generally, GAN models without conditional inputs can generate new images infinitely by adjusting the latent vector, whereas one input corresponds to one output image in typical conditional GAN. GAN techniques allow for more high-dimensional image transformation and realistic outputs than simple pixel-level transformation.

**Table 5:** Summary of literature review for domain transfer task using GAN in ophthalmology imaging domains

Publication	Basic technique	Domain	Summary
Costa et al. [58]	Conditional GAN	Vessel image → Fundus photography	The study proposed a vessel network to retinal image translation framework producing simplified vessel tree and realistic retinal images by estimating latent space. Autoencoder was used to synthesize new retinal vessel images apart from training of GAN
Zhao et al. [59]	Conditional GAN	Vessel image → Fundus photography	Retinal image synthesis can be effectively learned in a data-driven fashion from a relatively small sample size using a conditional GAN architecture
Yu et al. [60]	Pix2pix (with ResU-net generator) (conditional GAN)	Vessel image → Fundus photography	To enlarge training datasets for facilitating medical image analysis, the multiple-channels-multiple-landmarks (MCML) was developed to synthesize color fundus images from a combination of vessel and optic disc masked images
Wu et al. [61]	Conditional GAN	Volumetric retinal OCT → Fundus autofluorescence	The en-face OCT images were synthesized from volumetric retinal OCT by restricted summed voxel projection. The fundus autofluorescence images were generated from en-face OCT images using GAN to identify the geographic atrophy region
Tavakkoli et al. [62]	Conditional GAN	Fundus photography → Fluorescein angiography	The proposed GAN produced anatomically accurate fluorescein angiography images that were indistinguishable from real angiograms
Yoo et al. [63]	CycleGAN	Ultra-widefield fundus photography → Fundus photography	Ultra-widefield images were successfully translated into traditional fundus photography-style images by CycleGAN, and the main structural information of the retina and optic nerve was retained
Ju et al. [64]	CycleGAN	Fundus photography → Ultra-widefield fundus photography	The CycleGAN model transferred the color fundus photographs to ultra-widefield images to introduce additional data for existing limited ultra-widefield images. The proposed method was adopted for diabetic retinopathy grading and lesion detection
Lazaridis et al. [91, 108]	Wasserstein GAN + perceptual loss (conditional GAN)	Time-domain OCT → spectral-domain OCT	Time-domain OCT was converted to synthetic spectral-domain OCT using GAN. The model improved the statistical power of the measurements when compared with those derived from the original OCT

*GAN *= generative adversarial network; *OCT *= optical coherence tomography

Initially, Costa et al. demonstrated that a conditional GAN can be used to generate realistic fundus photographs guided by masked vessel network images [58]; an autoencoder was used to synthesize new retinal vessel images apart from training the GAN. Zhao et al. also built a conditional GAN model that emphasizes the ability to learn with a small dataset [59]. Extending the conditional GAN, modified Pix2pix synthesized realistic color fundus photographs to enlarge the image dataset based on multiple inputs of the vessel and optic disc masked images [60]. Wu et al. showed that retinal autofluorescence images could be synthesized based on retinal OCT data using a conditional GAN framework [61]. In that study, en-face OCT images from volumetric OCT data were successfully transformed into synthetic autofluorescence images to detect geographic atrophy regions in the retina. Tavakkoli et al. demonstrated that realistic retinal angiography images with diabetic retinopathy were generated via conditional GAN using fundus photographs [62]. Although the model was trained using a limited angiography dataset without detailed phase information, the study showed the potential of image domain transfer for the diagnosis of diabetic retinopathy. Based on CycleGAN, ultra-widefield fundus photography can be transformed into classic color fundus photography to integrate retinal imaging domains [63]. In contrast, a study converting classic fundus photography to ultra-widefield fundus photography via CycleGAN was also reported [64]. Larzaridis et al. demonstrated that time-domain OCT could be converted to spectral-domain OCT using conditional GAN with Wasserstein distance and perceptual loss, showing that the integrated dataset fused by the GAN improved the statistical power of the OCT measurements.

### Post-intervention prediction

The aim of post-intervention prediction is to generate an image that explains how the anatomical appearance changes after treatment. Three studies investigated post-intervention prediction tasks for ophthalmology imaging domains (detailed in Table 6). As post-intervention results are represented as images in several medical fields, this task is useful to clinicians and patients to understand how the intervention will affect the prognosis of diseases. However, the included studies have several limitations in terms of short-term follow-up periods for prediction and unstandardized interventions [65]. In addition, attention should be paid to interpreting the results because anatomical prediction after treatment is not necessarily related to functional outcomes such as visual acuity.

**Table 6:** Summary of literature review for post-intervention prediction task using GAN in ophthalmology imaging domains

Publication	Basic technique	Domain	Intervention	Summary
Yoo et al. [15]	Conditional GAN, CycleGAN	Periorbital facial images	Orbital decompression surgery	The developed model transformed preoperative facial input images into predicted postoperative images for orbital decompression for thyroid-associated ophthalmopathy
Liu et al. [66]	Pix2pix (conditional GAN)	Retinal OCT	Intravitreal anti-vascular endothelial growth factor injection	The model generated individualized post-therapeutic OCT images that could predict the short-term response of treatment for age-related macular degeneration
Lee et al. [67]	Conditional GAN (multi-channel inputs)	Retinal OCT (with fluorescein angiography and indocyanine green angiography)	Intravitreal anti-vascular endothelial growth factor injection	The trained model generated post-treatment optical coherence tomography (OCT) images of neovascular age-related macular degeneration

*GAN *= generative adversarial network; *OCT *= optical coherence tomography

Yoo et al. proposed a postoperative appearance prediction model for orbital decompression surgery for thyroid ophthalmopathy using a conditional GAN [15]. Although the experiment was performed at a relatively low resolution, the results show the potential of GAN as a decision support tool for oculoplastic and cosmetic surgeries related to the orbit. Two studies demonstrated that conditional GAN models could predict OCT images after anti-vascular endothelial growth factor (anti-VEGF) injection based on pre-injection OCT images with exudative age-related macular degeneration. Liu et al. showed that the Pix2pix model could generate synthetic post-injection OCT using pre-injection images to estimate the short-term response [66]. Lee et al. designed a conditional GAN with a multi-channel input for anti-VEGF injection [67]. The model was trained using both pre- and post-injection OCT images as well as fluorescein angiography and indocyanine green angiography to predict post-injection OCT.

### Feature extraction

Another task that did not belong to these categories was feature extraction, including out-of-distribution detection. This task focuses on the discriminative aspect of GAN because the studies have directly used GAN architectures to detect pathologies. Two studies have used the concept of GAN in ophthalmology image domains (Table 7). Schlegl et al. proposed an anomaly detection method using a GAN in the retinal OCT domain [25]. This GAN model estimated the latent space via inverse mapping learning from the input images and calculated anomaly scores from the feature space of normal samples. This anomaly detection architecture has been successfully extended to other areas, such as industrial anomaly detection or chest lesion detection in X-ray images [10]. Xie et al. built a modified conditional GAN model for ultra-widefield fundus photography to improve the detection of retinal diseases [26]. They used an attention encoder for feature mining in the generator and designed a multi-branch structure in the discriminator to extract image features.

**Table 7:** Summary of literature review for feature extraction task using GAN in ophthalmology imaging domains

Publication	Basic technique	Domain	Target	Summary
Schlegl et al. [25]	f-AnoGAN (Wasserstein GAN + latent space mapping)	Retinal OCT	Intra-retinal fluid detection (OCT anomaly detection)	The GAN based unsupervised learning of healthy training data was trained with fast mapping from images to encodings in the latent space. Anomalies were detected via a combined anomaly score based on an image reconstruction error
Xie et al. [26]	Conditional GAN (with attention encoder and multi-branch structure)	Ultra-widefield fundus photography (scanning laser ophthalmoscopy)	Features for retinal diseases	The GAN based on the attention encoder and multi-branch structure was used to extract features for retinal disease detection. The discriminator in GAN was modified to build the classifier to detect the disease images

*GAN *= generative adversarial network; *OCT *= optical coherence tomography

### Other applications

Here, we address several studies that did not fit our search criteria, but the applications are noteworthy. A localization task refers to the identification of a region of interest rather than a specific pixel segmentation. Zhang et al. performed retinal pathology localization in fundus photography using CycleGAN [68]. In this localization task, the pathological area was detected by subtracting the synthesized normal image from the pathological image.

Image registration is another task finding the geometric transformation to structurally align images. It is also important in automated analysis of multimodal image analysis such as domain transfer. For example, a dataset for conditional GAN requires additional image registration of aligned image pairs for successful training. Mahapatra et al. showed that an autoencoder based on GAN architecture provided better registration performance for fundus photography and retinal angiography images [69].

### Current limitations of GAN techniques

**Fig. 4:**
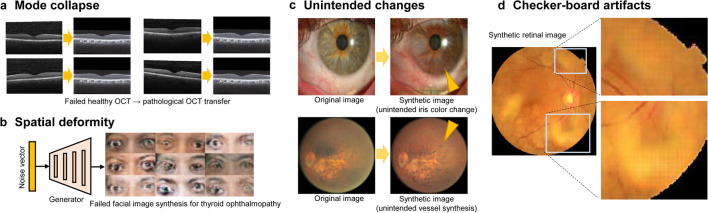
Examples of problems encountered using GAN techniques. **a** Mode collapse where the generator produces limited varieties of samples. **b** Spatial deformity due to small training images without spatial alignment. **c** Unintended changes due to the difference of data distribution between two domains. **d** Checker-board artifacts in synthetic images. All of the images were generated according to publicly available datasets and the standard GAN methods

We found that GAN has several limitations that researchers should take note of (Fig. 4). First, mode collapse, which is a phenomenon that continues to output the same results, is a well-known problem of GAN [70]. To avoid this failure caused by a model stuck in a local minimum, more variant training data or additional data augmentation techniques are needed. Many GAN models were trained with no guarantee of convergence. Second, spatial deformities frequently occur when there are small training images without spatial alignment. In particular, in domain transfer using conditional GAN, paired images with structural and spatial alignment are critically challenging and require additional image registration in a preprocessing to obtain high-quality medical images [57, 63]. Third, unintended changes could occur in image-to-image translation because of the different data distributions of the structural features between the two image domains. For example, if only one domain contains many images with glaucoma in a domain transfer task, CycleGAN can produce glaucomatous changes during image synthesis. Noise can be represented as unintended outliers/confounders and unintended fake features can be generated from noise for a generator in several GAN models [26]. Fourth, high-frequency noises and artifacts of checkerboard patterns are often detected in images synthesized by a generator with deconvolution [53]. Novel techniques have been developed to reduce noise and artifacts [71].

GAN and its variants generally consist of two or more deep learning modules, for example, two generators and two discriminators in CycleGAN, and thus training GAN tends to be unstable compared to a single deep learning module [15]. A problem of vanishing gradients may also occur if the discriminator performs well, and the generator learns too slowly. Therefore, tuning the hyperparameters is sometimes important, and training can be stopped early to obtain better synthetic images. However, the occurrence of these problems depends on the amount of data and the distribution of embedded pixels and is unpredictable.

In our experience, most of the problems from training GAN models are solved to some extent by increasing the amount of clinical data extracted from a variety of patients if the technique is appropriately selected. Although GAN is widely used for data augmentation, several previous studies using GAN for image-to-image translation also suffered from small amounts of data, similar to other deep learning algorithms [45]. As novel algorithms are emerging to solve these problems [72], GAN will eventually be easy to use for image processing in ophthalmology.

Additionally, there is no standard metric for evaluating the performance of GAN for realistic image synthesis [73]. It now relies on subjective judgments from researchers and clinicians relevant to ophthalmology imaging [40]. Previous studies adopted classic image similarity indices, such as mean squared error, mean absolute error, and structural similarity index [15], but they are unable to evaluate the realism of synthetic images. Several studies have shown an improvement in the diagnostic performance of machine learning after GAN-based data augmentation [43, 46], but it does not guarantee that GAN produces realistic images. This problem arises from the application of GAN not only in ophthalmology but also in all medical areas [10] and will continue to be a drawback for using GAN.

## Discussion

We surveyed the literature relevant to GAN in ophthalmology image domains to guide future studies on image processing in ocular images. To our knowledge, this work is the first comprehensive literature review on the use of GAN techniques in ophthalmology image domains. Recently, a review of GAN in ophthalmology was reported, but the scope was limited to image synthesis in fundus photography and OCT [73]. GAN research has thrived in the medical field because machine learning is *data-hungry* to achieve a more accurate diagnosis. In this review, we highlighted the various uses of GAN in that it can perform segmentation, data augmentation, denoising, domain transfer, super-resolution, post-intervention prediction, and feature extraction. The number of publications relevant to this field has also grown consistently as GAN techniques have become popular among researchers. We found that GAN can be applied to most ophthalmology image domains in the literature. As imaging plays a crucial role in ophthalmology, GAN-based image synthesis and image-to-image translation will be highly valuable in improving the quantitative and personalized evaluation of ocular disorders. Despite the increasing use of GAN techniques, we also found that it faces challenges for adaptation to clinical settings.

Recently, a previous paper suggested that the utility of GAN in image synthesis is unclear for ophthalmology imaging [73]. However, GAN techniques have shown better performance in the fields of radiology and pathology than other generative deep learning models, such as autoencoders, fully convolutional networks (FCNs), and U-nets [74, 75]. In the anomaly detection task for retinal OCT, GAN models including AnoGAN, Pix2pix, and CycleGAN outperformed a traditional autoencoder model, which simply learns latent coding of unlabeled image data [76]. FCN and U-Net are well-established deep generative models for detection and segmentation tasks for biomedical imaging domains [77]. As these do not consider the detailed features of the output images, the GAN framework can improve the image synthesis performance of the FCN and U-Net models [78]. A previous study on retinal vessel segmentation showed that conditional GAN outperformed U-Net and other generative techniques [28]. Given this trend, GAN is expected to improve image analysis technologies in various tasks. A more accurate comparison and benchmarking of GAN techniques will be enabled by future studies and more clinical data.

The proper selection of the GAN technique and statistical modeling of ocular imaging will improve the performance of each image analysis. In this review, we found that a broad range of custom architectures from GAN variants was used for different tasks. There is no evidence of a particularly superior GAN technique. Researchers can analyze ocular images by newly defining the custom objective functions of the GAN to fit the specific task and domain. For example, Cheong et al. modified the conditional GAN model to effectively denoise OCT images using a custom loss function including content, style, total variation, and shadow losses [16]. Moreover, researchers can incorporate prior information about each imaging domain to develop GAN models for specific tasks. For example, researchers have suggested several statistical distributions of retinal structures and noise modeling in OCT images [79]. Statistical modeling using prior information improved the performance of segmentation of retinal layers and detection of diabetic retinopathy in OCT [80]. Since GANs are also mathematical models for learning the statistical relationship of distributions of training and target data [6], statistical modeling using a prior domain knowledge is expected to improve GAN performance. To adopt this concept in GAN for medical imaging, various mathematical attempts and validations are needed in future studies.

Several studies have shown that GAN can be a good choice in overcoming data shortages and lack of large annotated datasets in ophthalmology [81]. Burlina et al. showed that a deep learning model trained with only synthetic retinal images generated by PGGAN performed worse than those trained with real retinal images (0.9706 *vs.* 0.9235 considering the area under the receiver operating characteristic curve) [40]. However, several studies have shown that machine learning models trained with data integrating both real and GAN-based synthetic images can outperform those trained with real images in retinal OCT [43], anterior segment OCT [82], ocular surface image [46], and corneal topography [47]. GAN was also used for data augmentation of OCT images with rare retinal diseases in a semi-supervised learning manner [45]. Studies have shown that GAN-based data augmentation can provide a tool to solve an over-fitting problem in imbalanced datasets owing to the lack of available pathological samples. The image synthesis ability of GAN also provides patient privacy because synthetic images preserve characteristics as they become unidentifiable. The synthetic data preserve the manifold in the feature space of the original dataset [83]. It might be possible that machine learning researchers release the synthetic dataset generated by GAN instead of a real dataset to demonstrate their model if there is a problem with patient privacy. Additionally, the annotation of the dataset can be incomplete and inaccurate because it is time-consuming and laborious. According to a previous report regarding cell segmentation in microscopy images, GAN can be a solution for this weak annotation problem [84].

Studies using image-to-image translation frameworks of GAN have focused on segmentation, domain transfer, denoising, super-resolution, and post-intervention prediction. The denoising function of the GAN may be effective in decreasing the effect of adversarial attacks in ophthalmology image domains [85]. Recently developed GAN architectures, such as Pix2pix and CycleGAN, have been widely applied in medical image domains [27]. However, these techniques require spatial alignment between the two image domains to obtain high-quality results. Therefore, additional image registration is required before the GAN performs a domain transformation [57]. If the structures in the images are not aligned, the GAN may perform an image-to-image translation with deformed results in synthetic images. In this review, we found that only one study performed image registration to align the retinal structures between classic fundus photography and ultra-widefield fundus photography to improve the performance of CycleGAN [63]. We anticipate that this image alignment issue for training GAN models will be highlighted when data from various centers measured from multiple devices are collected. More research is needed, but recent studies have shown that GAN can also provide solutions to this image alignment issue [69, 86].

As the deep learning techniques associated with GAN have been developed, the scope of medical image processing is rapidly expanding. For example, when GAN was first introduced, simple vessel segmentation was the most frequent application of GAN. The recent work of Tavakkoli et al. achieved a significant advancement in the retinal vessel segmentation problem because their conditional GAN model provides realistic retinal fluorescein angiography, which can be used in a clinical setting [62]. Novel image processing techniques using unpaired image datasets, such as CycleGAN, have extended the range of training image domains, so ocular images from more diverse modalities are expected to be used for developing AI systems [38, 63]. Multi-domain GAN models that handle images from various domains at once have also been developed to fuse data for more accurate diagnosis. For example, Lee et al. reported a conditional GAN using multi-domain inputs analyzing OCT and fluorescein angiography to predict more accurate post-treatment retinal state prediction [67]. In the future, new GAN techniques such as StyleGAN [13], which is excellent at extracting and transforming features, and StarGAN [23], which performs multi-domain transformation, are also expected to be used in the ophthalmology imaging domains to solve clinical problems. To adopt this rapid technical development, future studies require multidisciplinary (clinician–engineer) collaboration and collection of more multi-domain ocular images. Clinicians need to feedback to engineers to improve the technical completeness of GAN.

Most studies included in this review used training and validation datasets extracted from the same study group. We found that no clinical trials have been conducted that explored the use of GAN. Machine learning techniques, including GAN, do not guarantee performance in external datasets independent of training sets. It has not been confirmed whether data augmentation through GAN can increase the diagnostic accuracy of AI systems for ocular diseases in real clinics. A GAN can be used to bridge the domain gap between training and external data from different sources [64]. If difficult access to reliable annotated data from multiple data sources remains problematic, domain adaptation can be considered to address the generalization issue [87]. Domain adaptation via the domain transfer function of a GAN may provide a chance to use a machine learning system in different settings. For example, retinal images taken with ultra-widefield fundus photography can be analyzed by an AI system developed with FP via domain transfer using GAN [63, 64]. Although GAN has several shortcomings, its ability to adapt to domains and expand data by generating realistic images can increase generalizability and may help to increase the use of machine learning algorithms in ophthalmology image domains. However, further studies are required to determine whether the application of GAN techniques will improve the diagnostic performance of machine learning models in real world clinical situations.

## Conclusion

The findings of this work suggest that the direction of deep learning research in ophthalmology has benefited from GAN. GAN techniques have established an extension of datasets and modalities in ophthalmology. The adoption of GAN in ophthalmology is still in its early stages of clinical validation compared with deep learning classification techniques because several problems need to be overcome for practical use. However, the proper selection of a GAN technique and statistical modeling of ocular imaging will improve the performance of each image analysis. We hope that this review will fuel more studies using GAN in ophthalmology image domains. More accurate algorithms for the detection of pathological ophthalmic conditions would be enabled by selection of proper GAN techniques by maximizing the potential of ophthalmology datasets.

## Data Availability

Data sharing is not applicable to this article as no datasets were generated or analyzed during the current study.
